# Multiple Messaging Strategies for Increasing HPV Vaccination Intentions among English- and Spanish-Speaking Parents in the United States and Mexico

**DOI:** 10.3390/vaccines12060650

**Published:** 2024-06-11

**Authors:** Matthew S. McGlone, Keri K. Stephens, Mian Jia, Carolyn Montagnolo, Yifan Xu

**Affiliations:** 1 Technology and Information Policy Institute, Moody College of Communication, The University of Texas at Austin, Austin, TX 78712, USA; keri.stephens@austin.utexas.edu (K.K.S.); cmontagnolo@austin.utexas.edu (C.M.); yifan.xu@austin.utexas.edu (Y.X.); 2 Department of English, City University of Hong Kong, Hong Kong SAR 999077, China; james.mian.jia@cityu.edu.hk

**Keywords:** human papillomavirus, vaccination, text message reminders, social influence

## Abstract

The reported study compared the impact of four influence strategies (agency assignment, enhanced active choice, deviance regulation marking, and temporal framing) on English- and Spanish-speaking parents’ reported intention to vaccinate their children for HPV. An online experiment was conducted to examine the impact of the strategies. In a fractional factorial design, participating parents (N = 1663) were exposed to combinations of influence strategies in text messages presented as reminders they might receive from a healthcare provider about their child’s eligibility for the vaccine series. The results indicated small but significant impacts of agency assignment, enhanced active choice, and deviance regulation marking on parents’ reported vaccination intentions. The study adds to the research literature on HPV vaccination communication in two important respects. First, it demonstrated how incorporating evidence-based influence strategies into reminder messages can increase parents’ vaccination intentions, an important precursor and predictor of actual vaccine uptake. Second, it sets an important precedent by examining the effects of influence strategies on vaccination intentions across different languages.

## 1. Introduction

Human papillomavirus (HPV) infection is the leading cause of cervical cancer and is also implicated in certain anogenital, head, and neck cancers. Vaccines are available that can prevent infection by the two HPV strains (Types 16 and 18) most associated with cervical and anogenital cancers [1]. Although these vaccines are available in over 120 countries, first-dose vaccine coverage by age 15 among girls in these countries stalled at 20% in 2019 and declined to 15% in 2021 [2]. Uptake has been higher in wealthier countries but is still suboptimal. According to the 2021 US National Immunization Survey, there was a 61.7% series completion rate among US adolescents between 13 and 17, which is well below the *Healthy People 2030* objective of 80% [3].

Several approaches to improving vaccine completion rates have been studied, and the advice to date often focuses on healthcare providers recommending HPV vaccination and articulating its benefits to parents as soon as their children become eligible for it [4,5,6]. While this is a solid strategy, other message factors can improve uptake as well. For example, parent-directed text-message reminders have been shown to improve child HPV vaccination rates [7,8,9]. Typically, these messages inform parents about the dose due dates and/or scheduling details (clinic name, phone number, etc.) without employing influence strategies to encourage parents’ compliance [10]. However, health communication researchers have found that incorporating influence strategies into reminder messages can increase parents’ reported intentions to get their children vaccinated. For example, a simple influence strategy known as “agency assignment” significantly improved the persuasiveness of parent-directed HPV vaccination reminders in both English and Spanish. This strategy served to heighten parents’ concerns about the threat HPV poses to their children’s health and their reported intention to have their children vaccinated [11].

Agency assignment is just one of several evidence-based message features that can be used to promote HPV vaccination and can be incorporated easily into SMS messages and social media posts. The aim of the reported study was to gauge the impact of agency assignment and three other message features (active choice, deviance regulation marking, and temporal framing) on parents’ attitudes, beliefs, and intentions to vaccinate their children for HPV. An online message testing format with a fractional factorial design was employed to manipulate these features in the composition of vaccination reminder messaging and to measure outcomes that predict vaccination-seeking behavior.

### 1.1. Influence Strategies as Message Features

Researchers in behavioral economics, communication, social psychology, and related disciplines have investigated strategies to influence people’s beliefs, attitudes, and choices about health matters in ways that promote self-protective and pro-social behavior [12,13,14]. Although many of these strategies require background knowledge about, extended interaction with, and/or individual tailoring to target audience members, others can be implemented in relatively short and scalable interventions. The latter tend to involve strategic word choices that focus audience members’ attention on motives for enacting a desired behavior (e.g., the protection vaccination provides) rather than deterrents (e.g., concerns about vaccine efficacy or side effects).

These strategies are well-suited for vaccination reminders sent via text messaging. Several factors point to text messaging as a promising channel for increasing HPV vaccination uptake in the United States. First, studies of mobile technology usage indicate that approximately 80% of American adults own a smartphone, and 83% of them send/receive text messages daily. These regular texters spend an average of 26 min per day sending and receiving texts, open 99% of the texts they receive, and respond to 45% of received texts (in comparison to just 6% of emails), typically within 90 s [15]. Second, health interventions delivered via text messaging reach and engage disadvantaged and underserved groups more effectively than others disseminated through traditional channels [16]. Third, simple text message reminders notifying parents of their child’s eligibility for vaccination can be combined with other message features that encourage the initiation and completion of the vaccine series. Four evidence-based message influence strategies that were used for this purpose in the reported study are described below.

### 1.2. Agency Assignment

This linguistic feature describes the entity (person, object, or process) portrayed as performing action (and thereby assigned to the agent thematic role for the verb describing the action) in a sentence [17]. English, Spanish, and most other languages permit speakers to grammatically assign the agency for viral transmission to the virus (e.g., *The virus could infect millions of people*) or to people (*Millions of people could contract the virus*). Although they are often used interchangeably, these assignments shape different conceptions of transmission, as attributable to pursuit by an active predator or to social contact within one’s control [11]. Patient education materials and vaccination policy arguments about viral threats (COVID-19, H1N1, HPV, influenza, etc.) that consistently assign transmission agency to the virus have been shown to increase perceived health threat severity and vaccination intentions relative to materials assigning transmission agency to humans [11,18,19,20,21,22]. Consequently, we hypothesized that HPV vaccination reminder text messages assigning transmission agency to the virus would increase parents’ reported perceived viral threat severity and vaccination intentions relative to other messages assigning agency to humans or to a control message with no transmission agency assignment.

### 1.3. Choice Format

This feature derives from the “choice architecture” framework in behavioral economics developed by Nobel Laureate Richard Thaler [23,24]. Recommending a particular option in a choice scenario (e.g., choosing to vaccinate or not) typically entails describing the benefit of the recommended “default” option—e.g., *You can reduce your child’s risk of developing certain types of cancer by choosing to vaccinate them for HPV*. In contrast, “enhanced active choice” framing describes both the benefit of the recommended option and the cost of the non-recommended option—e.g., *You can reduce your child’s risk of developing certain types of cancer by choosing to vaccinate them for HPV or accept the higher risk by choosing not to*. While it may appear self-evident that foregoing the benefit of an option constitutes a loss of this benefit, making the cost explicit can affect choice because decision-makers usually do not contemplate the risks associated with the status quo unless prompted to do so [25]. In addition to strengthening the desirability of the recommended choice option, this message feature also discourages the chooser from perceiving either option as a default and thus encourages them to actively consider the consequences of both options. The effects of enhanced active choice framing of options have been demonstrated in studies of decision-making about charitable giving, retirement savings, value-based healthcare, healthy diet and exercise adherence, and seasonal flu vaccination [26,27,28]. Based on this research, we hypothesized that text message reminders employing the enhanced active choice framing for the decision to vaccinate would be associated with a higher reported intention of parents to vaccinate their children than traditional “default” framing that mentions only the benefits of vaccination or a control message with no choice wording.

### 1.4. Deviance Regulation Marking

A message’s influence on behavior depends critically on audience members’ perceptions of the social norms associated with the behavior. Although people are typically motivated to conform to social norms, their sense of individual identity derives from what they believe makes them unique. According to “deviance regulation theory” in social psychology, in situations where people perceive their behavior as highly relevant to their individual identity (e.g., when making important decisions), they are more sensitive to the costs and benefits of deviating from a perceived social norm than they are to conforming to it. Consequently, messages intended to influence their behavior should be more impactful when framed in terms of norm deviation rather than conformity [29]. For example, if a parent believes most other parents vaccinate their children for HPV, then the “unusual” risk associated with not doing this should be more salient for them than the benefits of doing what they believe most other parents do. On the other hand, if they think most other parents do not vaccinate their children, then they should be more attuned to the unusual benefit of doing it than the “common” risk associated with not doing it.

Deviance regulation theory has informed interventions designed to reduce binge-drinking and illegal drug use, to increase physical activity and safe sex practices, and to promote seasonal flu vaccination [30,31,32,33]. In the current study, we hypothesized that characterizing HPV vaccination as the norm and describing the risk of deviating from it would be associated with higher parent vaccination intentions than describing abstention as the norm and describing its benefits, or a control message with no mention of vaccination norms or consequences. This hypothesis is predicated on the assumption that a text message vaccination reminder from a healthcare provider would ostensibly cast vaccination as the social norm (both descriptive and injunctive) among our respondents rather than abstention.

### 1.5. Temporal Framing

Preventive behaviors involve a tradeoff between short-term costs and long-term benefits. Consequently, time is an important factor in people’s decision-making about health matters. Research indicates that present-oriented messaging focusing on immediate consequences tends to be more effective in encouraging people to perform a recommended health behavior than future-oriented appeals describing its consequences at a distant later time [34,35]. However, this general claim is qualified by certain characteristics of audience members and message format. In particular, individual differences in audience members’ proclivity for “consideration of future consequences” (CFC) have been shown to affect their receptivity to messaging about preventive behaviors (using sunscreen, colorectal screening, vaccination, etc.), such that low-CFC members are more persuaded by present-oriented messages and high-CFC members by future-oriented messages [36].

There is also evidence that temporal framing operates differently for persuasive stories (i.e., a sequence of events in which characters act and are acted upon) than didactic messages that directly explain and advise. In a study of HPV vaccination promotion messaging, it was found that testimonials of someone who had been vaccinated had a stronger impact on readers’ confidence in the vaccine and their intention to take it when they focused on short-term rather than long-term benefits; in contrast, messages that simply explained and recommended the vaccine were more impactful when focused on long-term rather than short-term benefits [37]. The researchers interpreted these findings in terms of “construal level theory” in social psychology, which posits that the remoteness of an event (socially, spatially, temporally, etc.) shapes how we think about it [38]. Events we consider remote are mentally represented abstractly, while proximate events are represented in more concrete terms. For example, an engaged couple having their wedding next year construes it abstractly as the “start of a new life,” but the couple doing this next week thinks about details such as having the flowers delivered on time, getting the pictures made, etc. [39]. Analogously, simply describing the cancer risk reduction benefit of HPV vaccination fits with the abstract construal induced by future-oriented framing, while a personal story about completing the series fits better with the concrete construal induced by present-oriented framing. Based on construal level theory, we hypothesized that future-oriented framing of benefits in vaccination text message reminders (which are intrinsically didactic) would be associated with a higher reported intention of parents to vaccinate their children than present-oriented framing or a control message with no temporal framing.

## 2. Materials and Methods

An online message testing experiment was conducted to test hypotheses about the effects of the aforementioned message factors on English- and Spanish-speaking parents’ reported intention to vaccinate their children for HPV. Because a full factorial design for testing all combinations of the 5 factors (language of message, agency assignment, enhanced active choice, deviance regulation marking, and temporal framing) would require a cumbersome set of 34 conditions (2^5^ experimental conditions + no-factor controls in English and Spanish), we employed a fractional factorial design strategy, which has been used in several studies of health messaging [40,41]. Fractional designs are most commonly used in “screening” studies conducted to determine which of a large set of candidate factors exert significant effects on outcome measures and thus merit further study [42]. The advantage of this strategy is that fewer conditions are required than in the full design. However, the advantage comes with a tradeoff in that the design can detect factor main effects but only a subset of the interactions between them [41,43]. We employed a resolution V fractional factorial design [44] with 18 conditions (16 experimental and 2 controls), detailed in Table 1.

### 2.1. Participants

1746 paid volunteers were recruited to participate in the study via the online audience panel platform *Centiment* (www.centiment.co; accessed on 2 March 2021). Volunteers were required to (a) be parents of a child 17 or under who had not been vaccinated for HPV, (b) be residents of the United States or Mexico, (c) have English or Spanish as their preferred language, and (d) own a mobile phone with text messaging and social media usage capacity. The rationale for including parents with children in the 17 and under range was to sample the spectrum of decision-making influences on parents’ vaccination decisions. Children in this particular age range vary significantly in their level of autonomy regarding health decisions. Adolescents are more likely to actively participate in or at least influence their vaccination decisions. In contrast, younger children typically rely more heavily on parental guidance for health-related choices [4,7,9].

The sample was stratified by preferred language in a 60–40 English–Spanish split, dictated by the availability of volunteers who met our participation requirements. English and Spanish-speaking volunteers were paid on average $2.75 and $3.50, respectively; payment varied individually by date of recruitment and time spent completing the study and across groups by representation in the available online population.

The choice of sample size was determined in part by power analytic considerations, with a targeted power of 0.85 for detecting predicted effects that would account for 5% of explained variance, applying two-tailed tests with a nondirectional α = 0.05. Another consideration was the prospect of having to eliminate data from participants who did not comply with instructions, as indicated by failure to correctly answer attention check questions. Based on these considerations, we recruited 10% more volunteers for the study than the 1620 required for adequate statistical power.

### 2.2. Message Stimuli

**Figure 1:**
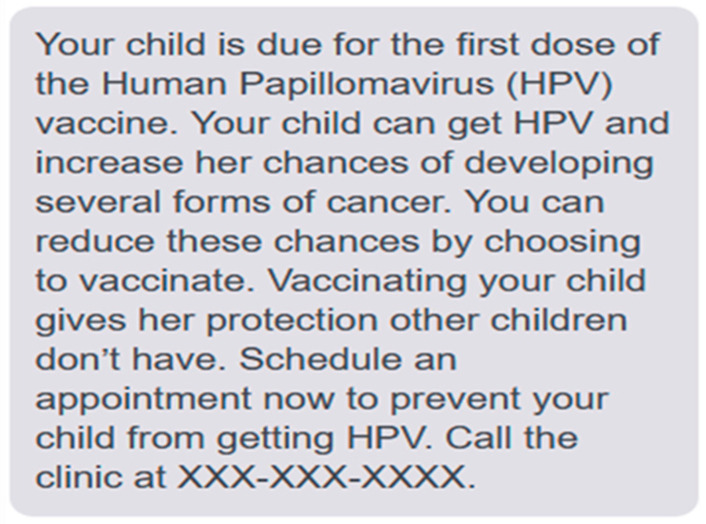
Example HPV vaccination reminder message.

The stimuli for this study were messages in English and Spanish notifying parents of their child’s eligibility for HPV vaccination and encouraging them to initiate the series. These messages were designed to be sufficiently brief for dissemination via an SMS-based vaccination reminder system [10]. Each message began with a sentence indicating the child’s eligibility (*Your child [name] is due for the first dose of the Human Papillomavirus (HPV) vaccine*) and ended with a sentence encouraging the parent to call the clinic (*Call the clinic at XXX-XXX-XXXX*). Control messages (in both English and Spanish) consisted only of these sentences, but the experimental messages also contained sentences with the aforementioned influence features presented in combinations listed in Table 1. Each experimental message contained one of two forms of each feature, as described below. An example experimental message is presented in Figure 1, and the full set of 18 (2 control and 16 experimental) stimulus messages is available in the Open Science Framework: https://doi.org/10.17605/OSF.IO/UR25C (accessed on 2 March 2021).

(1) *Agency Assignment:* “Virus agent” and “child agent” forms of this feature were created. The former attributed causality for HPV infection and its consequences to the virus itself (*HPV can infect your child and increase his/her/their chances of developing several forms of cancer*). The latter attributed infection causality to the child (*Your child can get HPV and increase his/her/their chances of developing several forms of cancer*).
(2) *Choice Format:* The “default” form of this feature described only the benefit of the recommended choice option (*You can reduce these chances by choosing to vaccinate*). The “enhanced active choice” form described both the benefit of the recommended option and the cost associated with the non-recommended option (*You can reduce these chances by choosing to vaccinate or accept the higher risk by choosing not to*).
(3) *Deviance Regulation Marking:* The “norm” form of this feature conveyed that choosing to vaccinate an eligible child is the most common option by describing the comparative risk of violating this norm (*Not vaccinating your child leaves him/her/them unprotected compared to other children*). The “deviation” form conveyed that vaccination was an uncommon option by describing the comparative benefit of deviating from this norm (*Vaccinating your child gives him/her/them protection other children don’t have*).
(4) *Temporal Framing:* The “present” form of this feature described the immediate benefit of vaccination (*Schedule an appointment now to prevent your child from getting HPV*). In contrast, the “future” form described the long-term benefit (*Schedule an appointment now to prevent your child from getting HPV-related cancers as an adult*).

### 2.3. Measures

Our attention, demographic, and psychological measures were incorporated into a questionnaire participants completed after reading one of the stimulus messages. Semantically equivalent English and Spanish versions of the questionnaire were created and used across experimental conditions. It consisted of 8 sections. Section 1 contained 4 questions (1 short answer and 4 multiple choice) designed to gauge participants comprehension of and attention to the presented message.

Sections 2–5 consisted of Likert-type items on a 7-point scale (1 = very strongly disagree to 7 = very strongly agree) measuring the perceived severity of the threat posed by HPV (3 items, α = 0.91; e.g., *HPV is dangerous*), self-efficacy regarding vaccination (3 items, α = 0.87; e.g., *There is nothing that would stop me from getting the HPV vaccine for my child if I want it*;), confidence in the vaccine (8 items, α = 0.89; e.g., *The HPV vaccine does a good job of preventing the diseases it is intended to prevent*), and intention to vaccinate (2 items, α = 0.86; e.g., *I intend to get my child vaccinated against HPV*), respectively. Items for the second, third, and fifth sections were adapted from measures used in prior work on vaccination messaging impact, and items in the fourth were adapted from the Vaccination Confidence Scale [45,46]. Perceived threat severity, self-efficacy, and vaccination confidence have been shown to predict HPV vaccination intention [18,20,22] and thus were included to gauge participants’ receptivity to stimulus message content. The two items measuring vaccination intention in Section 5 constitute our formal outcome measures [11,18].

Section 6 consisted of 4 items on a 5-point scale (1 = nothing at all to 7 = a lot), probing participants self-reported knowledge of the HPV virus and vaccine (α = 0.90; e.g., *How much do you know about the kinds of problems HPV can cause*?). Section 7 contained 8 multiple choice items that explored participants’ prior experience communicating with healthcare providers about the vaccine (e.g., *Has a healthcare provider talked with you about the HPV virus or its vaccine*?) and their direct experience with the vaccine (e.g., *Have you yourself been vaccinated against HPV*?). Finally, Section 8 contained a set of 12 standard short-answer and multiple-choice items about demographic characteristics (see Table 2) and ended with an open-ended question probing participants’ beliefs about the purpose of the study.

### 2.4. Procedure 

This study was registered before data collection in the *Open Science Framework*: https://osf.io/g6rsn/?view_only=968de8982d9a4453983f93a234de0789, accessed on 2 March 2021. Volunteers recruited by the crowdsourcing platform *Centiment* gained access to the *Qualtrics*-based online experiment via separate portals for English and Spanish speakers. After providing informed consent, participants were informed that the purpose of the study was to explore “how people read and interpret messages from their healthcare providers about the Human Papillomavirus (HPV) and its vaccine.” Next, they were reminded they had been recruited because they reported being the parent of a child who is 17 or younger and had not been vaccinated for HPV. For the purpose of personalizing the stimulus messages, they were given the option of providing the child’s name (or skipping this question) and asked to indicate the pronouns they used to refer to the child (*he/him/his, she/her/hers, they/them/theirs*). After providing this information, participants were randomly assigned to one of the 18 (9 English, 9 Spanish) message conditions.

The presented message was described as a “text message reminder that you might receive from a healthcare provider to arrange a vaccination appointment for your child.” It referred to the child by name if the participant provided it (otherwise referred to as “your child”), and the pronouns indicated. Participants were instructed to read the message carefully to prepare for questions about its content they would answer later on, and were informed it would appear on the screen for a minimum of 20 s before they could advance to the questionnaire. After completing the questionnaire, they were thanked for participating and directed back to the *Centiment* platform to claim payment. On average, participants spent 36 s (*SD* = 8.56) reading their assigned message and completed the entire experimental procedure in 9.77 min (*SD* = 1.31).

## 3. Results

Data were discarded from 83 of the 1746 (4.8%) participants who failed to answer at least two of the three multiple choice attention check items correctly. The sample size of the retained participants (*N* = 1663) was sufficient to meet our power target of 0.85. Data analyses were conducted in *IBM SPSS* 28.0. A demographic profile of the sample is provided in Table 2. The majority of respondents were female (65.0%), White (78.3%), non-Hispanic (55.1%), and U.S. residents (82.1%) who spoke English as their preferred language (58.4%) and had an average age of 37.9 years (*SD* = 8.22). Most also reported having completed an associate or more advanced college degree (55.0%), being married (63.6%), being employed full time (57.4%), and living in a household with an annual income at or below the US median of $70K (55.7%). Although the majority reported that their unvaccinated child had a regular doctor or other healthcare provider (84.4%) and that they had talked with their healthcare provider about the HPV vaccine (54.5%), a minority reported having been vaccinated personally (44.6%) or having one or more other children who had been vaccinated (45.2%). A plurality of respondents reported referring to their child using *he/him/his* pronouns (49.3%), followed by *she/her/hers* (47.8%), and *they/them/theirs* (2.8%). A randomization check revealed no significant associations between any of the aforementioned sociodemographic or health-related variables and assignment to experimental conditions, with *p* > 0.20 in all cases.

Table 3 presents means, standard deviations, and Pearson correlations for the psychological scale variables. Mean scores for all 5 variables were above their scale midpoints, indicating a general trend for respondents to report planning to have their child vaccinated (vaccination intention; *M* = 5.12, *SD* = 1.47 on a scale of 1 to 7) and to perceive the HPV vaccine as safe and effective (vaccine confidence; *M* = 4.99, *SD* = 1.12), to believe they were capable of getting their child vaccinated (self-efficacy; *M* = 5.35, *SD* = 1.33), to consider themselves as knowledgeable about the virus and vaccine (HPV knowledge; *M* = 3.13, *SD* = 0.93), and to view the threat to health posed by HPV as severe (health threat severity; *M* = 5.48, *SD* = 1.48). In addition, all five of the variables were significantly positively correlated, with *p* < 0.01 in all cases. The reliable positive associations observed among these variables are consistent with prior studies of the psychological predictors of vaccination [11,20,45,46].

Hierarchical multiple regression was employed to determine the degree to which measured and manipulated variables explained the variance in participants’ reported vaccination intentions. Variables were entered into the regression in four blocks. Sociodemographic variables (the first 10 variables in Table 2) were entered in the first block, health-related variables (the last 4 in Table 2) were added in the second, psychological variables (the last 4 in Table 3) were added in the third, and the manipulated message variables (agency assignment, choice format, deviance regulation, and temporal framing) and their second-order interactions with language (language*agency assignment, language*choice format, language*deviance regulation, and language*temporal framing) were added in the fourth. Note that our use of a fractional factorial design did not permit us to include interactions among the message variables (e.g., agency assignment*choice format) as predictors because only a subset of the combination conditions were represented in our design. Table 4 presents statistics associated with each block model.

Diagnostics were computed to determine whether any multicollinearity problems existed in our regression analysis [47]. Inspection of the variance inflation factors (VIFs) generated for each variable indicated that none exceeded 4, a conservative criterion for variable removal [48]. However, the VIFs for preferred language and Hispanic status were outliers (2.62 and 2.30, respectively) and, not surprisingly, these variables were highly correlated, *r* (1661) = 0.75, *p* < 0.001. Both variables were retained in the analysis, but we acknowledge the possibility that their retention might have resulted in modest variance inflation.

The results indicated that the first model explained significant variance in vaccination intention, with *F* (10, 1651) = 14.47, *p* < 0.001, *R*^2^ = 0.081. Significant predictors in this model included respondents’ preferred language (*β* = 0.21, *t* = 5.58, *p* < 0.001), education (*β* = 0.06, *t* = 2.11, *p* < 0.035), and household annual income (*β* = 0.06, *t* = 2.39, *p* < 0.017). Inspection of the vaccination intention ratings indicated that Spanish-speaking respondents generally reported stronger intentions (*M* = 5.56, *SD* = 1.25) than English-speaking respondents (*M* = 4.81, *SD* = 1.53). These ratings were also modestly positively correlated with both education level, *r* (1661) = 0.124, *p* < 0.001, and household annual income, *r* (1661) = 0.088, *p* = 0.005. Adding health-related variables in the second model (*F* (13, 1648) = 13.85, *p* < 0.001, *R*^2^ = 0.099) significantly increased the variance accounted for over the first model, *∆F* (3, 1648) = 10.91, *p* < 0.001, *∆R*^2^ = 0.018. As in the first model, significant predictors in the second model included the sociodemographic variables primary language (*β* = 0.16, *t* = 4.21, *p* < 0.001), education (*β* = 0.05, *t* = 2.09, *p* = 0.037), and household annual income (*β* = 0.07, *t* = 2.76, *p* = 0.006).

Three health-related variables also explained significant variance: communication with a healthcare provider about HPV and the vaccine (*β* = 0.09, *t* = 3.68, *p* < 0.001), the parent’s vaccination status (*β* = 0.09, *t* = 3.56, *p* < 0.001), and whether or not the parent had other children who had been vaccinated (*β* = 0.08, *t* = 2.77, *p* = 0.006). Inspection of the vaccination intention ratings indicated that responding parents who reported having talked with a healthcare provider about HPV and the vaccine expressed stronger intentions to get their child vaccinated (*M* = 5.21, *SD* = 1.38) than others who had not (*M* = 5.06, *SD* = 1.52) or were not sure (*M* = 4.88, *SD* = 1.57). In addition, parents who reported having personally received the HPV vaccine series expressed stronger intentions to have their children vaccinated (*M* = 5.43, *SD* = 1.30) than others who had not (*M* = 4.81, *SD* = 1.60) or were not sure (*M* = 5.20, *SD* = 1.05). Finally, parents who reported having another child who had been vaccinated for HPV expressed stronger intentions to have their currently eligible child vaccinated (*M* = 5.40, *SD* = 1.31) than others who did not (*M* = 4.87, *SD* = 1.57) or were not sure (*M* = 5.19, *SD* = 1.13).

Adding psychological variables in the third model (*F* (17, 1644) = 204.13, *p* < 0.001) significantly and substantially increased the variance accounted for over the second, *∆F* (4, 1644) = 741.63, *p* < 0.001, *∆R*^2^ = 0.580. As in the first two models, preferred language was a significant predictor (*β* = 0.06, *t* = 2.52, *p* = 0.012), but no other sociodemographic nor health-related variable explained significant variance, with *p* > 0.10 in all cases. However, two psychological variables were significant predictors: perceived health threat severity (*β* = 0.06, *t* = 3.49, *p* < 0.001) and vaccine confidence (*β* = 0.81, *t* = 44.38, *p* < 0.001). As indicated in Table 3, vaccination intention ratings were positively correlated with perceptions of the severity of the health threat posed by HPV, *r* (1661 = 0.36, *p* < 0.001, and with vaccine confidence, *r* (1661) = 0.82, *p* < 0.001. It is notable that the large effect size for vaccine confidence is consistent with other studies investigating determinants of intention to seek vaccination for HPV and other pathogens [49,50].

Adding the manipulated message variables in the fourth and final model (*F* (25, 1636) = 168.99, *p* < 0.001, *R*^2^ = 0.689) significantly increased the variance accounted for over the third model, ∆*F* (8, 1636) = 4.28, *p* < 0.001, ∆*R*^2^ = 0.010. As in the first three models, preferred language was a significant predictor (*β* = 0.06, *t* = 2.41, *p* = 0.016), but no other sociodemographic nor health-related variable explained significant variance, *p* > 0.10 in all cases. As in the third model, two psychological variables were significant predictors: perceived health threat severity (*β* = 0.06, *t* = 3.59, *p* < 0.001) and vaccine confidence (*β* = 0.81, *t* = 44.60, *p* < 0.001). Three of the manipulated message variables were also significant predictors: agency assignment (*β* = 0.04, *t* = 2.24, *p* = 0.025), choice format (*β* = 0.04, *t* = 2.13, *p* = 0.033), and deviance regulation (*β* = 0.09, *t* = 4.95, *p* < 0.001). However, temporal framing was not a significant predictor (*β* = 0.01, *t* = 0.82, *p* = 0.412), nor were any of the interactions between preferred language and the message variables, with *p* > 0.15 in all cases. The fact that these interaction terms were not significant predictors indicates that the manipulated message variables did not appear to influence English- and Spanish-speaking respondents in appreciably different ways.

Inspection of the vaccination intention ratings indicated that the significant effects exerted by the message variables on intention were consistent with our hypotheses. Vaccination reminder messages assigning transmission agency to the virus elicited higher intention ratings (*M* = 5.20, *SD* = 1.46) than other messages assigning agency to the child (*M* = 5.08, *SD* = 1.49) or the control message (*M* = 4.94, *SD* = 1.47). Messages employing the “enhanced active choice” format elicited higher intention ratings (*M* = 5.23, *SD* = 1.44) than others employing the default format (*M* = 5.07, *SD* = 1.46) or the control message. Finally, messages casting vaccination as a social norm and criticizing deviation from it elicited higher ratings (*M* = 5.28, *SD* = 1.42) than others casting abstention as the norm and praising deviation from it (*M* = 5.00, *SD* = 1.47), or the control message.

## 4. Discussion

There is a growing body of evidence demonstrating that parent-directed text message reminders can improve HPV vaccine series initiation and completion rates for eligible children. This evidence comes from prospective cohort studies, randomized controlled trials, and pragmatic clinical trials [51,52,53,54,55,56,57,58]. The current study adds to this research literature in three important respects. First, it explored how incorporating evidence-based influence strategies in reminder messages can increase parents’ vaccination intentions, an important precursor and predictor of actual vaccine uptake. Although a few studies have examined the effects of individual influence strategies on vaccination intentions, only one of the strategies under study here (agency assignment) has been previously examined in reminder messages, and, to the best of our knowledge, no prior study has examined multiple messaging strategies in the same research design. Second, we set another important precedent by examining the effects of influence strategies on the vaccination intentions of both English- and Spanish-speaking respondents. Third, our research design enabled us to determine the extent to which usage of each messaging strategy explained variance in vaccination intention above and beyond sociodemographic, health-related, and psychological characteristics of the respondents.

Three sociodemographic variables—preferred language, education, and household income—explained significant variance in vaccination intention in the first two models. These findings are consistent with previous studies of vaccine uptake that have employed only sociodemographic variables as predictors [5,59]. The only sociodemographic variable to explain significant variance in vaccination intention in the full model was preferred language, with Spanish-speaking respondents reporting higher intentions to vaccinate their children than English speakers. This finding might seem counterintuitive in light of prior studies indicating that Hispanic/Latinx individuals have among the highest HPV infection rates, and Hispanic/Latinx parents’ are less likely to have their children vaccinated for HPV than other ethnic groups. However, there is considerable variability in estimates of HPV vaccine uptake in this population, with some recent studies indicating their uptake to be among the highest of any ethnicity in the United States [59,60]. One potential explanation for higher uptake in this population is a heightened sense of susceptibility attributable to higher cervical cancer and mortality incidence in Hispanic/Latinx communities [61]. Moreover, other recent surveys have found that Hispanic/Latinx adolescents whose parents’ preferred language was Spanish had higher vaccination rates than others whose parents’ preferred English [62,63,64]. Language preference is highly correlated with measures of acculturation (with preference for Spanish among U.S. residents indicating lower acculturation) and is often treated as a proxy for these measures [65]. Given the frequent finding that lower acculturation is associated with higher uptake, our results are consistent with this pattern. However, we only measured parents’ reported intentions to vaccinate rather than actual uptake, and various barriers (access to medical care, transportation, etc.) can hinder parents from following through on their vaccination intentions [61,63,66].

Two psychological variables emerged as significant predictors: the perceived severity of the health threat posed by HPV and vaccine confidence. Both are important factors in models of how audiences process persuasive appeals about health and wellness. Arguably the most prominent is the extended parallel process model (EPPM), which posits that audience members decide to change their behavior in response to a health challenge based on separate appraisals of the threat it poses to their health and their capacity to meet the challenge. According to the model, the degree to which the audience member feels threatened by the challenge determines their motivation to act, while their sense of efficacy in addressing the threat determines the action they take. Our findings are consistent with EPPM predictions regarding perceived health severity and response efficacy (i.e., confidence in the proposed solution for addressing the health threat, vaccination in this case) and with several other studies of vaccination intention [67,68,69,70]. However, the model presumes that the efficacy appraisal includes consideration of both response efficacy and self-efficacy (i.e., confidence in one’s ability to enact the response), the latter of which did not emerge as a significant predictor of vaccination intention in our study. One potential explanation for this finding is that getting a child vaccinated is an easy health behavior to enact compared to others such as ensuring they exercise regularly or eat a healthy diet; consequently, self-efficacy may have been generally too high (*M* = 5.35 on a 7-point scale) to account for significant variance in intention.

Three of the four manipulated message variables emerged as significant predictors of vaccination intention: agency assignment, choice format, and deviance regulation. Although their effect sizes were much smaller than for vaccine confidence, it is nonetheless notable that they were comparable to the effect sizes for other sociodemographic and psychological predictors and that these effects derived from a single message exposure. Respondents who read reminder messages assigning transmission agency to the virus (*HPV can infect your child*) indicated higher intentions to vaccinate their children than others who read messages assigning agency to the child (*your child can get HPV*). This finding is consistent with previous studies demonstrating a persuasive advantage for messages assigning threat agency to HPV and other pathogens such as COVID-19, H1N1, and antibiotic-resistant bacteria [11,18,19,20,21,22,71]. It also comports with evolutionary accounts of human fear, suggesting threats are prioritized when perceived as exogenous and autonomous [72].

Choice format also influenced vaccination intentions, as predicted. Specifically, reminder messages describing both the benefit of vaccination and the cost of abstention prompted respondents to report higher intentions than messages describing only vaccination benefits. According to behavioral economists, the advantage of the former format derives from cuing the decision-maker to anticipate the regret they might experience if they forego the recommended action and the cost (in this case, their child developing an HPV-related cancer) comes to pass [25]. This finding comports with several demonstrations of the “enhanced active choice” format’s influence on people’s decision-making in both health and economic contexts [26,27,28].

Our hypothesis about the effects of deviance regulation marking on vaccination intentions was also confirmed. Reminder messages that characterized HPV vaccination as the norm and described the risks of deviating from it elicited higher reported intentions than others that characterized abstention as the norm and described the benefits of deviating from it. According to deviance regulation theory, individuals’ motivation to engage in a health behavior is determined by what they perceive as the norm regarding that behavior, whether the norm is descriptive (i.e., what people generally do), injunctive (i.e., what people should do), or both [29]. In the context of a message from a healthcare provider recommending that parents have their children vaccinated for HPV, vaccination (as opposed to abstention) is clearly framed as an injunctive norm and may also have been perceived as the descriptive norm. Under these circumstances, respondents were motivated to avoid deviating from the norm and thereby reported higher vaccination intentions.

Finally, our hypothesis regarding the temporal framing of the benefit of vaccination was not supported. Specifically, future-oriented framing of the benefit (i.e., reducing the child’s likelihood of developing an HPV-related cancer as an adult) did not prompt respondents to indicate higher vaccination intentions than present-oriented framing (reducing the child’s likelihood of contracting HPV). There are several possible reasons for this finding. First, the long-term potential of HPV to cause forms of cancer was mentioned in the experimental messages as part of the agency assignment manipulation. Consequently, respondents could have been able to infer vaccination’s long-term benefit of reducing the chances of cancer even if the benefit had not been explicitly stated. Second, several individual difference factors (e.g., cultural conceptions of time) have been found to moderate how people respond to temporally framed messages across multiple outcomes [33,34,35,36,37]. These factors were neither measured nor controlled in the current study, and as a result, their potentially moderating influence on our findings could not be assessed. Third, evidence for the benefit of future-oriented over present-oriented framing in expository messaging indicates its effect size to be small, and thus it is possible the benefit was simply too slight and/or fragile to be detected in the current study [73].

### Study Strengths and Limitations

The strengths of this study include its large and diverse sample (ethnically and linguistically), the use of sociodemographic and psychological measures to distinguish between variance accounted for by individual differences and by message factors, and the manipulation of multiple messaging strategies in the same research design. There are also several limitations. First, we relied on an online convenience sample without population weighting, which restricted our ability to interpret the findings as indicating predictors and message effects generalizable to national or international populations. In particular, our sample was highly educated and generally reported high vaccination intentions, and consequently, our participants’ comprehension of and receptivity to the reminder messages might not generalize to parents with less education and/or a lower inclination toward vaccination a priori. Second, our dependent measure was parents’ reported intention to vaccinate their children for HPV rather than actual uptake. Although parents’ intention is an important precursor and predictor of vaccine uptake, a 2018 meta-analysis of studies measuring both variables [63] indicated the association between them is modest (*r* = 0.31, 95% CI [0.17, 0.43]). Consequently, the observed message reminder effects on parents’ vaccination intentions cannot be directly generalized to impacts on the initiation and/or completion of the HPV vaccine series. Third, intention in our study was measured within minutes of exposure to one of the text message reminders, and so it is possible their effects on intention were short-lived. Fourth, the reminders were not delivered to respondents’ mobile phones by their healthcare providers but rather presented in the contrived context of an online survey. Thus, it is possible respondents might have interpreted and evaluated reminder content differently in a more naturalistic context.

Our use of a fractional factorial experimental design constitutes both a strength and a weakness. This design strategy is primarily used in screening experiments, in which the objective is to test multiple factors and determine which exert significant effects and thereby merit further study [41]. For our purposes, the strength of this strategy was its informational efficiency, in that it allowed us to test multiple message factors with fewer conditions and a smaller sample than would be required by a full factorial design. However, including only a subset of all possible factor combinations in the study limited our ability to explore interactions between them. The only factor interactions we explored were between preferred language and influence strategies, none of which emerged as significant predictors of vaccination intention. Nevertheless, our findings indicated that 3 of the 4 influence strategies tested (agency assignment, choice format, and deviance regulation marking) exerted significant main effects on participating parents’ intentions to vaccine their children for HPV. These effects occurred for both English- and Spanish-speaking respondents and were of comparable effect size. Future research is necessary to determine the duration of these effects, their potential impact on vaccine uptake, and potential interactions among the influence strategies.

## 5. Conclusions and Practical Implications

Parent-directed text message reminders have been shown to improve HPV vaccine series initiation and completion rates for eligible children. Our findings suggest that the impact of these reminders on parents’ vaccination intentions can be augmented by the inclusion of evidence-based influence strategies. Specifically, we found that reminders were most effective in increasing participating parents’ reported vaccination intentions avwhen they (a) assigned HPV transmission agency to the virus, (b) described both the benefits of vaccination and the costs of abstention, and (c) characterized vaccination as a social norm and referred to the risks of deviating from this norm.

## Figures and Tables

**Table 1:** Fractional factorial design of the message testing experiment.

Condition	Language	Agency Assignment Assignment	Choice Format	Deviance Marking RE Regulation Marking	Temporal Framing
1 (Control)	English	None	None	None	None
2	English	Virus	Enhanced	Norm	Future
3	English	Virus	Enhanced	Deviation	Present
4	English	Virus	Default	Norm	Present
5	English	Virus	Default	Deviation	Future
6	English	Child	Enhanced	Norm	Present
7	English	Child	Enhanced	Deviation	Future
8	English	Child	Default	Norm	Future
9	English	Child	Default	Deviation	Present
10 (Control)	Spanish	None	None	None	None
11	Spanish	Virus	Enhanced	Norm	Future
12	Spanish	Virus	Enhanced	Deviation	Present
13	Spanish	Virus	Default	Norm	Present
14	Spanish	Virus	Default	Deviation	Future
15	Spanish	Child	Enhanced	Norm	Present
16	Spanish	Child	Enhanced	Deviation	Future
17	Spanish	Child	Default	Norm	Future
18	Spanish	Child	Default	Deviation	Present

**Table 2:** Demographic characteristics of the sample (N = 1663).

Characteristic	n	%
Preferred Language		
English	972	58.4
Spanish	691	41.6
Country of Residence		
United States	1365	82.1
Mexico	298	17.9
Age		
18–29	266	15.9
30–39	785	47.2
40–49	403	24.2
50–59	189	11.4
60–69	20	1.2
Gender		
Female	1082	65.0
Male	552	33.2
Transgender	26	1.6
Other/Prefer Not to Answer	3	0.2
Race		
African–American	89	5.4
American Indian/Alaskan Native	19	1.1
Asian	48	2.9
Biracial	48	2.9
Native Hawaiian or Other Pacific Islander	7	0.4
White (including Hispanic Whites)	1302	78.3
Other	150	9.0
Culturally or Ethnically Hispanic		
No	916	55.1
Yes	747	44.9
Marital Status		
Married	1058	63.6
Not married but in a committed relationship	273	16.4
Separated	82	4.9
Divorced	103	6.2
Widow/Widower	20	1.2
Never Married	127	7.6
Education		
Less than High School Degree	30	1.8
High School Degree or equivalent	310	18.6
Some College but no degree	409	24.6
Associate’s Degree (2-year undergraduate college)	164	9.9
Bachelor’s Degree (4-year undergraduate college)	525	31.6
Master’s Degree	176	10.6
Doctorate or Professional Degree	49	2.9
Employment		
Full-Time	954	57.4
Part-Time	235	14.1
Student	18	1.1
Unemployed and looking for work	122	7.3
Unemployed and not looking for work	123	7.4
Other	173	10.4
Household Annual Income		
Less than $20,000	287	17.3
$20,000–$39,000	347	20.9
$40,000–$59,000	291	17.5
$60,000–$79,000	227	13.7
$80,000–$99,000	165	9.9
$100,000–$119,000	121	7.3
$120,000 or more	225	13.5
Child Has Regular Doctor or Other Healthcare Provider		
No	259	15.6
Yes	1404	84.4
Parent Talked with Healthcare Provider about HPV Vaccine		
No	711	36.1
Yes	907	54.5
Not Sure	45	6.6
Parent Personally Vaccinated for HPV		
No	800	48.1
Yes	742	44.6
Not Sure	121	7.3
Parent Has 1 or More Children Vaccinated for HPV		
No	866	52.1
Yes	752	45.2
Not Sure	45	2.7

**Table 3:** Means, standard deviations, and correlations (with 95% confidence intervals) for scale variables.

Variable	*M*	*SD*	1	2	3	4
1. Vaccination Intention	5.12	1.47				
2. Vaccine Confidence	4.99	1.12	0.82 **			
3. Self-Efficacy	5.35	1.33	0.45 **	0.56 **		
4. Virus and Vaccine Knowledge	3.13	0.93	0.15 **	0.19 **	0.11 *	
5. Health Threat Severity	5.48	1.48	0.36 **	0.49 **	0.59 **	0.15 **

* *p* < 0.01, ** *p* < 0.001.

**Table 4:** Hierarchical multiple regression analysis predicting vaccination intention from measured and manipulated variables.

Model/Significant Predictors	*F*	*β*	*t*	*p*	*R*	*R* ^2^	*DR* ^2^
1. Sociodemographic Variables	*F (10, 1651) = 14.47, p < 0.001*				*0.284*	*0.081*	*0.081*
Preferred Language		*0.21*	*50.58*	*<0.001*			
Education		*0.06*	*20.11*	*0.035*			
Household Annual Income		*0.06*	*20.39*	*0.017*			
2. Sociodemographic + Health Variables	*F (13, 1648) = 13.85, p < 0.001*	*0.31*	*0.099*		*0.314*	*0.099*	*0.018*
Preferred Language		*0.16*	*40.21*	*<0.001*			
Education		*0.05*	*20.09*	*0.037*			
Household Annual Income		*0.07*	*20.76*	*0.006*			
Parent Talked with Provider about HPV		*0.09*	*30.68*	*<0.001*			
Parent Vaccinated		*0.09*	*30.56*	*<0.001*			
Parent Has Other Vaccinated Children		*0.08*	*20.77*	*0.006*			
3. Sociodemographic + Health + Psychological Variables	*F (17, 1644) = 204.13, p < 0.001*				*0.824*	*0.679*	*0.580*
Preferred Language		*0.06*	*20.52*	*0.012*			
Vaccine Confidence		*0.81*	*440.38*	*<0.001*			
Perceived Health Threat Severity		*0.06*	*30.49*	*<0.001*			
4. Sociodemographic + Health + Psychological + Message Variables	*F (25, 1636) = 168.99, p < 0.001*				*0.830*	*0.689*	*0.010*
Preferred Language		*0.06*	*20.411*	*0.016*			
Vaccine Confidence		*0.81*	*440.60*	*<0.001*			
Perceived Health Threat Severity		*0.07*	*30.59*	*<0.001*			
Agency Assignment		*0.04*	*20.24*	*0.025*			
Choice Format		*0.04*	*20.13*	*0.033*			
Deviance Regulation Marking		*0.09*	*40.95*	*<0.001*			

Note: *β* = standardized regression coefficient; *F* = Fisher’s F test; *t* = Student’s t test; *p* = Type I error probability; *R* = multiple correlation; *R*^2^ = coefficient of determination; *∆R*^2^ = *R* square change.

## Data Availability

The data reported in this article are available in an Open Science Framework repository: https://doi.org/10.17605/OSF.IO/UR25C; accessed on 24 August 2023.
